# Pneumatization within a maxillary sinus graft: a case report

**DOI:** 10.1186/s40729-015-0036-9

**Published:** 2016-01-19

**Authors:** Viviane Rozeira Crivellaro, João César Zielak, Tatiana Miranda Deliberador, Naylin Danyele de Oliveira, Felipe Rychuv Santos, Carmen Lucia Mueller Storrer

**Affiliations:** Universidade Positivo, Curitiba, PR Brazil

**Keywords:** Maxillary sinus, Pneumatization, Bone graft vascularization, Angiogenesis

## Abstract

Rehabilitation of edentulous posterior maxilla with implant-supported prostheses frequently presents a challenge to dentists. This is due to insufficient bone within the region, in addition to other limiting factors such as anatomical pneumatization of the maxillary sinus. Thus, grafting of the maxillary sinus is a common procedure used to counteract these problems. Regardless of the type of biomaterial used, the success of the procedure is dependent on the formation of high-quality bone. Therefore, vascularization is a key factor for successful grafting and for the long-term maintenance of the treatment. This paper reports a clinical case of bone graft pneumatization and attempts to elucidate its potential etiology.

## Background

The application of osseointegrated dental implants has being increasingly used for functional and esthetic rehabilitation of partially or completely edentulous patients [1]. However, to ensure eligibility for this type of procedure, the patient must exhibit a sufficient amount of bone in the region to be reconstructed. This is because insufficient bone height or thickness contraindicates the rehabilitation of patients using implants [2]. Patients who have experienced total or partial early dental loss require bone remodeling, and severe bone loss by resorption will typically occur. Resorption is caused by several processes, including the lack of physiological stimulation of the periodontal ligaments and the exertion of pressure by the removable prosthesis [3].

The anatomy of the posterior region of the maxilla limits the installation of the implant due to the presence of the maxillary sinuses and limited bone density in this region [4]. Resorption and bone remodeling in this region can result in pneumatization of the maxillary sinus. Consequently, the height of the alveolar crest to the floor of the sinus becomes insufficient for the placement of dental implants with satisfactory stability under masticatory forces [5]. Sinus graft surgery can increase bone height and is therefore the initial procedure of choice for correction of this problem.

Currently, several types of biomaterials can be utilized as bone substitutes. In addition to the autogenous graft, a procedure still considered the “gold standard” of bone grafting [6], allografts, alloplastic implants, xenogenous implants, or a combination of more than one type of biomaterial may also be used. The success of the procedure, regardless of the type of biomaterial used, is dependent on the formation of a vascular network inside the filler material. Sufficient vascularization is a prerequisite for the formation of bone of adequate quality [7]. Various angiogenic factors interact during the induction of endothelial cells to form new vessel; endothelial cells act as the main mediator in neovascularization [8]. Irrigation of grafted biomaterial by adjacent vessels is crucial and, through diffusion, provides sufficient nutrients and modulators for the revascularization amidst the biomaterial particles. Therefore, it is likely that angiogenesis process disorders will lead to the inhibition of new bone formation.

Herein, we report a case of pneumatization within a maxillary sinus graft and review the literature for potential etiologies. To our knowledge, no similar clinical cases have been reported in the literature.

## Case presentation

A non-smoking 59-year-old woman with a total absence of upper teeth and a complete prosthesis in this region sought medical care at the Dentistry Department of the Universidade Positivo. The patient’s main complaint was a difficulty in eating because of an ill-adapted upper prosthesis. She requested the installation of dental implants and a protocol-type prosthesis.

**Fig. 1:**
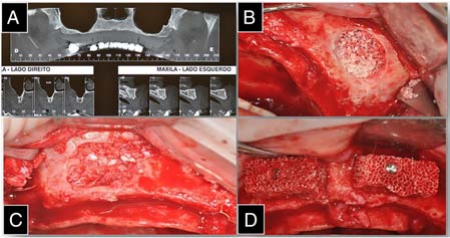
**a** Computed tomography (CT) was requested to plan the procedure. **b** Lyophilized particulate human bone administered into the right maxillary sinus. **c** Mineralized granular bovine bone was administered into the left maxillary sinus. **d** Two blocks of mineralized bone inserted in the anterior region of the maxilla

Computed tomography (CT) was requested to plan the procedure. The analysis of the digital examination revealed that both the left and right maxillary sinuses were pneumatized and that the anterior region had an extremely thin flange making implant surgery impossible owing to reduced bone thickness (Fig. 1a). Therefore, it was evident that surgery was required for the installation of osseointegrated implants in the maxilla. Grafting of the maxillary sinuses (to increase the height) and block grafts in the anterior region (to increase thickness) were proposed to correct these defects. A second phase was required for the surgical implantation of the implants.

On the day of the surgery, after asepsis and anesthesia, a supracrestal incision from the region of the right maxillary tuberosity to the left maxillary tuberosity was performed. Osteotomy was performed to create an accession window in order to access the right and left maxillary sinuses, under abundant sterile saline irrigation. The membranes of the right and left maxillary sinuses were detached. During this procedure, the Schneiderian membrane was accidentally perforated in the right sinus, and therefore, a bovine biological barrier was inserted (GenDerm, Baumer, Mogi-Mirim, São Paulo, Brazil).

Lyophilized particulate human bone, acquired from the Bone Bank of the Hospital das Clínicas de Curitiba (Curitiba, Paraná, Brazil), was mixed with saline and administered into the right maxillary sinus until thoroughly compacted. Mineralized granular bovine bone was administered on the left side (OrthoGen, Baumer, Mogi-Mirim, São Paulo, Brazil), which was also mixed with physiological saline and compressed (Fig. 1b, c), and postoperative care were passed to the patient.

**Fig. 2:**
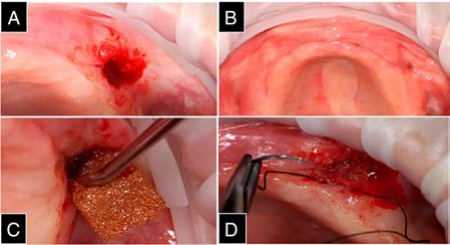
**a** Communication between the left maxillary sinus and the oral cavity (oroantral communication). **b** Epithelization of the area with the insertion of a fibrin sponge impregnated with colloidal silver. **c** The sponge was glued to the edges of the flap. **d** The bloody tissue was sutured

Three months after surgery, a reassessment was performed, and clinical examination revealed a communication between the left maxillary sinus and the oral cavity (oroantral communication) (Fig. 2a). An epithelization of the area was conducted together with the insertion of a fibrin sponge impregnated with colloidal silver (Gelatamp, Roeko, Langenau, Germany) (Fig. 2b). The sponge was glued (Glubran 2, GEM S. r. l, Viareggio [LU], Italy) to the edges of the flap (Fig. 2c), and the bloody tissue was sutured with nylon (5-0, Shalon, São Luiz de Montes Belos, Goiás, Brazil) (Fig. 2d). Following this, medication and postoperative care were prescribed. After 15 days, when the patient returned for a follow-up visit for suture removal and clinical examination, the oroantral communication had closed.

**Fig. 3:**
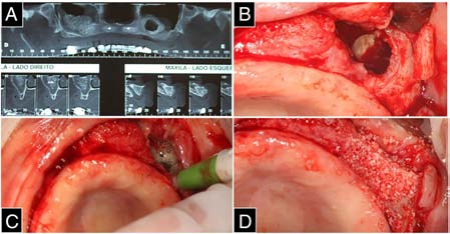
**a** CT with a circular image suggesting potential cyst formation. **b** Open-access window without any secretion at the site. **c** The site was irrigated using saline with added antibiotics. **d** The pneumatization site of the bone graft was re-filled with granular bovine bone biomaterial

Three months later (6 months postoperatively), a new CT was requested. A circular image within the graft of the left maxillary sinus was observed, suggesting potential cyst formation (Fig. 3a). One month after the second CT, the patient underwent biopsy surgery for histological analysis of the interior content of the suspected cyst. A supracrestal surgical incision was performed with full detachment of the flap, exposing the mounting bolts of the anterior blocks and providing access to the left maxillary sinus. After the flap was folded, the left maxillary sinus still presented with an open-access window and lacked filling material. There was no presence of odor or secretion at the void site, which made it impossible to carry out the biopsy (Fig. 3b).

The graft was completely pneumatized, with walls surrounding the void, mimicking the formation of an additional maxillary sinus within the existing one. Side, top, and bottom walls were present. There was not any type of epithelium inside the bony walls. The site was irrigated using saline with added antibiotics (a supernatant of 500 mg of tetracycline dissolved in 10 mL of saline) after confirming that there was no communication with the rest of the maxillary sinus (Fig. 3c).

**Fig. 4:**
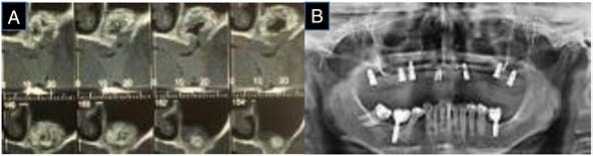
**a** CT slices (oroantral communication). **b** Panoramic radiograph (follow-up)

After irrigation, the pneumatization site of the bone graft was re-filled with granular bovine bone biomaterial (Fig. 3d). Simple suturing was performed for cooptation of the flap edges, followed by a continuous suture, again as an attempt to close the oroantral communication, which can be observed in detail in the CT slices (Fig. 4a). The patient was administered similar postoperative patient care as that administered earlier.

One year later, after another bone grafting, seven implants were performed in the patient's maxilla for her prosthetic rehabilitation (Fig. 4b).

### Discussion

In order to determine the potential cause of the pneumatization within the maxillary sinus graft reported in this case, various factors influencing the success of bone remodeling must be considered.

Later stage complications such as wound dehiscence, oroantral fistula, and postoperative maxillary cysts (mucocele) are relatively common [9]. A small oroantral fistula usually closes spontaneously if the patient is medicated with antibiotics and chlorhexidine solution. In the present case, the presence of an oroantral communication was observed 3 months postoperatively. The patient was not aware of this communication and was only informed of this during the clinical review. Therefore, since a spontaneous closure was not possible in this case, surgical measures for its correction were planned, along with administration of antibiotics.

Since the patient was submitted to a CT scan for treatment planning, a new CT scan was indicated only after 6 months postoperatively, for biosafety reasons [10]. This initial diagnosis of a cyst was rational, since cysts are common after stimulation or inflammation of the maxillary sinus [9]. However, the graft was completely pneumatized, with hard walls surrounding the void, mimicking the formation of an additional maxillary sinus within the existing one, but without any Schneiderian membrane—which made it difficult for the collection of a sample for an histopathological differential diagnosis. Upon surgical access of the suspected cyst, it was possible to confirm that a hollow cavity in which only the peripheral particle material inserted as a maxillary sinus filler was present. Regarding this matter, it can be assumed that there was insufficient irrigation to allow cell migration to the center of the graft. Therefore, this region lacked the defense and nutrition necessary for the maintenance of tissue volume. In other words, the lack of angiogenesis or vasculogenesis in the central region of the graft, a key part to new bone formation, led to this pneumatization within the graft.

Any type of local infection can compromise grafts, and this likely occurred in the present case, generating purulent material that was expelled by a fistular process, finding an easy route into the oral cavity, leading to oroantral communication. One factor that may have influenced the lack of neovascularization is that the patient had a widespread vitamin deficiency, which was found after blood analyses request. Another important factor is that the patient, despite having omitted during the interview, was an alcoholic. Alcoholic patients are generally more prone to failure in bone grafting surgery [11].

Three months after the graft surgery of maxillary sinus, as there was a perceived oroantral communication, the insertion of a fibrin sponge was performed. As pneumatization occurred only on the left side, it could be suggested that the cause of angiogenesis absence could be influenced by the insertion of the sponge in the oroantral communication closing operation. However, Gelatamp® is used for treatment of wounds, since colloidal silver acts releasing silver ions, which act against a broad spectrum of bacteria [12]. Furthermore, the use of topical silver has a popular widespread use for being effective against infections [13]. In our case, its use was intended to speed up the healing and closure of the oroantral communication, favoring the process of cure and prophylaxis of wound. Also, the sponge has hemostatic properties [14], and for this reason, it is used after dental extraction, acting as a scaffold in the alveolus for the installation and proliferation of cells. Within this scaffold, the environment would be favorable and conducive to bone formation and would not prevent the formation of new bone; therefore, the sponge could not have prevented the vascularization of the area. In this case, a minimum piece of sponge was placed solely to assist in healing of the epithelium and connective tissue. For the replacement of bone graft biomaterial by natural bone, an invasion of the osteoblasts accompanied by the formation of new vessels is required [7]. This neovascularization is an essential prerequisite for the formation of new bone of sufficient quality [15] and it may be accomplished with the use of sponge with colloidal silver, which perhaps was not large enough in this case to exercise that function. The bone cells lie within a complex matrix composed of a mineralized component (hydroxyapatite) and a non-mineralized component (predominately collagenous). The non-mineralized component (comprising approximately 30 % of the matrix) is responsible for the growth and differentiation of osteoblasts, osteocytes, and osteoclasts, in addition to bone remodeling [16]. In this component, a large number of angiogenesis regulatory factors interact in a coordinated manner to induce the formation of vessels [17]. The diffusion of fluids and blood perfusion is key for the successful formation of new bone. Therefore, in the present case, it can be suggested that angiogenesis was insufficient for the induction of new bone formation, leading to the formation of a pseudocyst.

In support of this, one report in 1996 [18] proposed several requirements for successful bone grafts. These included the presence of osteoblasts on site, sufficient blood supply for graft nutrition, material stability, and the maintenance of soft tissue closure by suturing without tension. Therefore, the survival of any type of grafting cell relies on processes such as the diffusion of fluids and blood perfusion. In the reported case, tissues were coopted adequately without tension, the material was stable, and the presence of osteoblasts was likely inhibited by a lack of nutrition. In addition to angiogenesis, vasculogenesis can also result in the formation of new blood vessels. Angiogenesis occurs through the branching of pre-existing blood capillaries, while vasculogenesis takes place through the differentiation of undifferentiated endothelial cells [19]. Vascularization of the grafted area begins with the migration and proliferation of endothelial cells. These cells form a network of new bone capillaries because one of the functions of these cells is to regulate angiogenesis. In the present case, bone maturation was not evident in the center of the grafted area where vascularization was most difficult to occur.

The initial process of angiogenesis is diffusion. However, diffusion can only occur at a distance of up to a maximum of 200 μm in the matrix. Therefore, for large tissue reconstruction, the survival of the central reconstruction cells is limited by initial vascularization difficulties [20]. Consequently, due to limited initial vascularization, diffusion into the center of large tissue growth is limited making it difficult for the survival of more centralized cells [21]. Thus, it can be concluded that angiogenesis is essential for successful bone grafting. In the present case, it is likely that angiogenesis did not occur in the center of the graft, leading to the pneumatization of the bone graft in grafted maxillary sinus.

## Conclusions

According to the literature and the present case report, it can be concluded that the greater the requirement for tissue reconstruction, the greater is the dependence on maintenance processes and cellular growth. For sufficient graft development, particularly those involving exogenous biomaterials, one of the most important processes is vascularization. In the present case, successful completion of the sinus graft was initially compromised, probably due to limited blood supply. This led to the center of the graft receiving insufficient nutrition causing subsequent collapse and pneumatization within the grafted biomaterial.

## Consent (Adult)

Written informed consent was obtained from the patient for the publication of this report and any accompanying images.
